# Socio-economic and health-related determinants of unmet health needs: a national cross-sectional study on the adult population

**DOI:** 10.3325/cmj.2025.66.35

**Published:** 2025-02

**Authors:** Vesna Štefančić Martić, Ana Ivičević Uhernik, Tomislav Benjak, Petra Čukelj, Ivana Brkić-Biloš, Danijela Štimac Grbić

**Affiliations:** Croatian Institute of Public Health, Zagreb, Croatia

## Abstract

**Aim:**

To investigate the prevalence and predictors of self-perceived unmet health needs (UHN) in Croatia.

**Methods:**

We used data from the European Health Interview Survey, conducted in 2019 on 5461 individuals. Dependent variables were different causes of UHN – long waiting times, financial problems, and problems with transportation, while independent variables were sex, age, marital status, region of residence, degree of urbanization of the respondent's place of residence, education level, quintile of household's income, self-perceived general health, self-reported chronic disease/condition, severity of bodily pain, consultation with a family doctor in the last 12 months, consultation with a specialist in the last 12 months, and perceived social support. An association between the variables was determined with a simple logistic regression and multiple logistic regression model.

**Results:**

Socioeconomic variables such as low education, urban residence, and residence in the Adriatic Region, as well as several health-related factors (worse self-perceived health, higher perceived levels of bodily pain, consultation with a physician in the last 12 months, and lower perceived levels of social support) were associated with higher odds for unmet health needs.

**Conclusion:**

Our findings highlight the need for continued reform and targeted initiatives in the Croatian health care system to improve health care access and equity. Addressing the underlying causes of UHN can help to ensure fair health care for all individuals and enhance overall health outcomes.

An unmet health need (UHN) is a situation in which someone with a perceived need for health care did not receive it. Obstacles to accessing health care can stem from individual preferences, financial constraints, or the unavailability of services. This article examines socio-economic and health-related determinants of UHN due to their impact on health care access and utilization. Socio-economic aspects, such as income, education, and employment, shape an individual's capacity to afford and navigate health care systems, while health-related factors, such as chronic illnesses and disabilities, influence the need for medical care and the obstacles to obtaining timely treatment (1-4). By analyzing these determinants, our study sought to offer an insight into both structural and individual factors that contribute to UHN.

The characteristics of a health care system can significantly affect its accessibility. The Croatian health care system is based on the principles of universality and solidarity (5). The Law on Health Care ensures the right to equal health care for equal health needs. The Croatian Health Insurance Fund provides two different health insurance packages – mandatory health insurance (6) and voluntary complementary health insurance. Access to services involves co-payments. These copayments were relatively small before 2008 but have since then increased several times, affecting system accessibility. However, private health insurance companies offer plans that can cover the co-payments and provide better accommodation standards (7-9).

The Croatian health care system has experienced numerous reforms in the last thirty years. Although it is still based on the principles of universal health care, these transitions might have partly disrupted access to health care (7). Previous studies showed that the Croatian public negatively perceives the consequences of health care reform in Croatia (8-10).

Due to a lack of studies on UHN in Croatia so far, there are no available data on how these changes in health care system might have influenced UHN in the population. This research sought to evaluate a range of factors related to different types of UHN in Croatia using data from the European Health Interview Survey. The primary aim was to identify the predictors of UHN caused by waiting lists, transportation or distance challenges, and financial barriers to medical examinations or treatments.

## METHODS

The analysis used data from a third wave of the European Health Interview Survey (EHIS). The sample was derived through a stratified two-stage random sampling of private households. Out of 3600 selected dwellings, the study involved 2580 households, encompassing 5461 individuals aged 15 and above. Trained community nurses interviewed participants in their homes between April and December 2019. All analyses were performed on weighted data, ensuring that the results are representative of the adult population in Croatia.

Three aspects of self-perceived UHN were analyzed separately as dependent variables: long waiting lists, problems with transportation, and financial problems. For all three dependent variables, participants who answered “yes” were compared with those who answered “no,” which meant that they had all their health care needs met during the last 12 months.

Independent variables included sex, age, marital status, region of residence, degree of urbanization of the respondent's place of residence, education level, quintile of household income, self-perceived general health, self-reported chronic disease/condition, severity of bodily pain, consultation with a family doctor in the last 12 months, consultation with a specialist in the last 12 months, and perceived social support (according to Oslo-3 Social Support Scale).

The regions of residence were either Adriatic or Continental, according to the Nomenclature of Territorial Units for Statistics 2016 classification. The level of urbanization was categorized based on the Classification of Local Administrative Units by Degree of Urbanization (Degree of Urbanization-Background). For this study, cities, towns, and suburbs were combined into a single category, referred to as urban areas.

The educational attainment level (highest completed) was classified according to the International Standard Classification of Education 2011.

Household income quintiles were calculated using the data on household size and composition and the total net monthly income of a household. For each household, the equalized net monthly income was derived by dividing the household total net monthly income by the adjusted size of the household.

In terms of perceived general health, respondents were divided into those reporting very good or good health, fair health, and bad or very bad health. According to self-reported chronic disease/condition, respondents were divided into those who reported such a disease/condition as present in the past 12 months and those who did not. The severity of bodily pain was classified into four categories: none, mild, moderate, and severe. Respondents were categorized into those who reported consultation with a general practitioner (GP) in the last 12 months and those who did not. The same was applied for consultation with a specialist. Perceived social support was classified as poor, intermediate, or strong according to the main categories of the Oslo-3 Social Support Scale.

### Statistical analysis

Descriptive statistics were computed separately for each of the listed reasons for self-perceived unmet needs across all independent variables. Logistic regression was conducted to assess the relationship between self-perceived UHN (dependent variable) for each reason and various predictor variables (independent variables), in comparison with the respondents whose health care needs were met. The relationship between each predictor variable and the dependent variable was assessed using a simple logistic regression (without adjusting for other predictors) and a multiple logistic regression model developed through a backward stepwise elimination process. The analysis was conducted with SPSS Statistics, version 27 (IMB Corp., Armonk, NY, USA).

Self-reported UHN can be presented in two ways: as a percentage of the entire population, regardless of whether the population members had any health care needs in the previous period, or as a percentage of those who reported having had health care needs within a specified timeframe. In this study, we reported the percentages of unmet needs among individuals who reported having had health care needs in the previous 12 months.

## RESULTS

Out of all respondents who reported any health care need in the previous 12 months, 40.1% had UHN due to any reason (95% CI 38.1%-42.0%), 26.3% had unmet health care needs due to too long waiting time (95% CI 24.7%-28.0%) (Table 1), 7.8% due to problems with transportation (95% CI 6.8%-8.8) (Table 2), and 17.8% due to financial reasons (95% CI 16.4%-19.4%) (Table 3).

**Table 1:** The prevalence of unmet needs due to too long waiting time according to various demographic and socioeconomic characteristics

	Self-perceived unmet health care needs due to too long waiting time			
Variables	n	%	95% CI	Pearson χ2 test (*P* value)
**Sex**				0.007
male	408	23.6	21.2-26.1	
female	655	28.3	26.0-30.6	
**Age (years)**				<0.001
15-34	114	18.5	14.9-22.6	
35-44	93	23.6	19.1-28.8	
45-54	153	25.2	20.9-29.9	
55-64	214	25.8	22.3-29.6	
65-74	252	33.4	29.3-37.7	
75+	237	29.0	25.3-32.9	
**Marital status**				0.001
never married and never in a registered partnership	141	20.9	17.3-25.1	
married or in a registered partnership	633	25.5	23.5-27.7	
widowed or in registered partnership that ended with death of partner	204	29.7	25.6-34.0	
divorced or in registered partnership that was legally dissolved	71	36.6	28.3-45.7	
**Region**				0.754
Continental	724	26.1	24.1-29.2	
Adriatic	339	26.7	23.9-29.7	
**Place of residence**				<0.001
urban	655	28.2	26.0-30.5	
rural	408	22.3	20.2-24.5	
**Education**				0.722
primary or less	307	25.7	22.8-28.9	
secondary	565	27.0	24.7-29.4	
higher than secondary	183	25.4	21.6-29.7	
**Net monthly equalised income of the household (quintiles)**				0.099
1st (poorest)	149	31.0	26.4-36.0	
2nd	147	29.0	24.7-33.8	
3rd	146	30.9	26.0-36.3	
4th	131	28.4	23.5-34.0	
5th (richest)	119	22.9	18.7-27.7	
**Self-perceived general health**				<0.001
very good and good	334	18.3	16.2-20.6	
fair	468	31.4	28.5-34.3	
bad and very bad	261	39.8	35-2-44.5	
**Self-reported chronic diseases/conditions**				<0.001
yes	778	32.5	30.2-34.9	
no	278	17.9	15.7-20.4	
**Severity of bodily pain**				<0.001
none	213	16.7	14.3-19.4	
mild	357	26.3	23.4-29.3	
moderate	275	31.9	28.1-36.0	
severe	217	43.2	38.0-48.6	
**Consultation with a family doctor in the last 12 months**				<0.001
yes	976	29.3	27.4-31.3	
no	85	12.9	10.1-16.4	
**Consultation with a specialist in the last 12 months**				<0.001
yes	777	34.7	32.3-37.2	
no	282	16.3	14.2-18.5	
**Perceived social support (Oslo-3 Social Support Scale)**				<0.001
poor support	171	31.5	26.8-36.5	
intermediate support	497	28.9	26.3-31.6	
strong support	380	22.1	19.7-24.6	

**Table 2:** The prevalence of unmet needs due to problems with transportation

	Self-perceived unmet health care needs due to problems with transportation			
Variables	n	%	95% CI	Pearson χ2 test (*P* value)
**Sex**				0.084
male	117	6.7	5.5-8.3	
female	208	8.5	7.2-10.0	
**Age (years)**				<0.001
15-34	24	3.9	2.4-6.1	
35-44	23	5.8	3.7-9.1	
45-54	41	7.7	5.2-11.2	
55-64	55	5.6	4.1-7.6	
65-74	83	10.8	8.5-13.7	
75+	99	11.1	8.8-14.0	
**Marital status**				0.001
never married and never in a registered partnership	42	6.4	4.4-9.3	
married or in a registered partnership	162	6.6	5.5-7.8	
widowed or in registered partnership that ended with death of partner	95	12.2	9.6-15.3	
divorced or in registered partnership that was legally dissolved	19	9.2	5.3-15.7	
**Place of residence**				0.942
urban	183	7.8	6.6-9.2	
rural	142	7.7	6.4-9.2	
**Region**				0.011
Continental	193	6.8	5.8-8.0	
Adriatic	132	9.5	7.8-11.6	
**Education**				<0.001
primary or less	144	11.8	9.7-14.1	
secondary	139	6.3	5.2-7.6	
higher than secondary	39	6.7	4.6-9.5	
**Net monthly equalized income of the household (quintiles)**				0.327
1st (poorest)	54	9.9	7.4-13.1	
2nd	52	10.3	7.6-13.7	
3rd	42	9.3	6.4-13.2	
4th	38	6.5	4.5-9.3	
5th (richest)	29	7.6	4.9-11.4	
**Self-perceived general health**				<0.001
very good and good	75	3.8	2.9-5.0	
fair	128	8.7	7.1-10.5	
bad and very bad	122	17.7	14.4-21.5	
**Self-reported chronic diseases/conditions**				<0.001
yes	246	9.9	8.5-11.4	
no	77	5.0	3.8-6.5	
**Severity of bodily pain**				<0.001
none	48	4.3	3.1-6.1	
mild	95	6.5	5.2-8.2	
moderate	84	8.7	6.8-11.0	
severe	98	19.2	15.3-23.7	
**Consultation of a family doctor in the last 12 months**				0.002
yes	292	8.5	7.4-9.7	
no	33	4.5	3.0-6.6	
**Consultation of a specialist in the last 12 months**				0.001
yes	212	9.4	8.1-11.0	
no	112	5.9	4.7-7.3	
**Perceived social support (Oslo-3 Social Support Scale)**				<0.001
poor support	76	13.4	10.4-17.0	
intermediate support	146	8.1	6.7-9.7	
strong support	101	6.0	4.7-7.6	

**Table 3:** The prevalence of unmet needs due to financial reasons

	Self-perceived unmet health care needs due to financial reasons			
Variables	n	%	95% CI	Pearson χ2 test (*P* value)
**Sex**				0.045
male	292	16.0	14.1-18.3	
female	452	19.1	17.1-21.3	
**Age (years)**				0.003
15-34	87	12.0	9.4-15.3	
35-44	66	16.8	12.8-21.7	
45-54	100	19.1	14.8-24.2	
55-64	153	16.7	13.8-20.0	
65-74	150	18.9	15.9-22.4	
75+	188	22.5	19.1-26.3	
**Marital status**				0.322
never married and never in a registered partnership	115	15.6	12.6-19.2	
married or in a registered partnership	433	17.5	15.6-19.5	
widowed or in registered partnership that ended with death of partner	145	19.8	16.3-23.9	
divorced or in registered partnership that was legally dissolved	39	21.1	14.4-29.7	
**Place of residence**				<0.001
urban	473	19.9	17.9-22.0	
rural	271	13.6	11.9-15.4	
**Region**				0.151
Continental	477	17.0	15.3-18.9	
Adriatic	267	19.3	16.9-22.0	
**Education**				0.287
primary or less	247	20.1	17.4-23.1	
secondary	375	17.1	15.3-19.2	
higher than secondary	118	17.2	13.7-21.3	
**Net monthly equalised income of the household (quintiles)**				0.951
1st (poorest)	122	20.4	17.0-24.3	
2nd	108	18.8	15.3-22.8	
3rd	104	19.5	15.7-24.0	
4th	93	20.1	16.0-25.0	
5th (richest)	81	18.4	13.9-24.0	
**Self-perceived general health**				<0.001
very good and good	254	13.4	11.5-15.6	
fair	279	18.7	16.3-21.3	
bad and very bad	209	30.7	26.6-35.2	
**Self-reported chronic diseases/conditions**				<0.001
yes	487	20.7	18.7-22.9	
no	254	14.3	12.4-16.5	
**Severity of bodily pain**				<0.001
none	160	11.1	9.2-13.3	
mild	258	18.6	16.0-21.5	
moderate	169	21.4	18.1-25.0	
severe	156	30.1	25.4-35.3	
**Consultation of a family doctor in the last 12 months**				0.205
yes	613	18.4	16.9-20.1	
no	129	15.7	12.3-19.7	
**Consultation of a specialist in the last 12 months**				0.167
yes	402	18.9	17.0-21.1	
no	336	16.8	14.7-19.1	
**Perceived social support (Oslo-3 Social Support Scale)**				0.001
poor support	131	23.5	19.6-27.9	
intermediate support	331	19.1	16.9-21.6	
strong support	268	15.0	12.8-17.5	

The prevalence of UHN due to too long waiting times and due to financial reasons was higher among women than among men, although 95% CIs slightly overlapped in both cases. Older age groups (65+ years) compared with the age group 15-34 years had a higher prevalence of UHN for all three reasons. Widowed or divorced participants compared with the never-married group had a higher prevalence of UHN due to problems with transportation and financial reasons. Urban dwellers compared with rural dwellers had a higher prevalence of UHN due to too long waiting times and financial reasons. The residents of the Adriatic compared with the Continental region had a higher prevalence of UHN due to problems with transportation, although 95% CIs slightly overlapped. Those with primary education or less compared with those with tertiary education had a higher prevalence of UHN due to problems with transportation. Respondents with bad self-perceived health, self-reported chronic diseases/conditions, and severe bodily pain had a significantly higher prevalence of all three aspects of UHN. Those who consulted a family doctor or a specialist in the last 12 months had a higher prevalence of unmet needs due to too long waiting time and problems with transportation than those who did not. Finally, those with poor self-perceived social support had higher prevalence of all three aspects of unmet needs compared with those with strong support (Tables 1,2,3). Sex, age, marital status, urban/rural place of residence, self-perceived general health, self-reported chronic diseases/conditions, bodily pain, consultation with a GP or a specialist in the last 12 months and self-perceived social support were all significantly associated with unmet needs due to too long waiting times. However, after controlling for other factors using a multiple logistic regression model, the variables that remained significantly linked to unmet needs due to long waiting times were urban or rural residence, self-rated general health, bodily pain, consultation with a family doctor or specialist in the past 12 months, and self-perceived social support. Rural dwellers had a lower adjusted odds ratio for unmet needs than urban dwellers. Conversely, respondents who reported poor health, severe bodily pain, recent consultations with a family doctor or specialist, and inadequate social support had higher adjusted odds ratios for unmet needs due to long waiting times (Table 4).

**Table 4:** Self-perceived unmet health care needs due to too long waiting times

	Self-perceived unmet health care needs due to too long waiting times			
Variable	OR unadjusted	95% CI	OR multivariable-adjusted**	95% CI
**Sex**				
male	1.00			
female	1.28	1.07-1.52		
**Age (years)**				
15-34	1.00			
35-44	1.37	0.94-1.98		
45-54	1.49	1.05-2.11		
55-64	1.54	1.12-2.11		
65-74	2.21	1.61-3.04		
75+	1.80	1.31-2.47		
**Marital status**				
never married and never in a registered partnership	1.00			
married or in a registered partnership	1.29	1.00-1.68		
widowed or in registered partnership that ended with death of partner	1.59	1.17-2.17		
divorced or in registered partnership that was legally dissolved	2.18	1.40-3.39		
**Region**				
Continental	1.00			
Adriatic	1.03	0.86-1.24		
**Place of residence**				
urban	1.00		1.00	
rural	0.73	0.62-0.87	0.75	0.63-0.90
**Education**				
primary or less	1.00		1.00	
secondary	1.07	0.87-1.30	1.31	1.05-1.64
higher than secondary	0.99	0.75-1.29	1.35	0.99-1.83
**Net monthly equalized income of the household (quintiles)**				
1st (poorest)	1.00			
2nd	0.91	0.66-1.25		
3rd	1.00	0.72-1.39		
4th	0.89	0.63-1.25		
5th (richest)	0.66	0.47-0.93		
**Self-perceived general health**				
very good and good	1.00		1.00	
fair	2.04	1.67-2.49	1.53	1.21-1.93
bad and very bad	2.95	2.31-3.77	1.64	1.21-2.22
**Self-reported chronic diseases/conditions**				
no	1.00			
yes	2.20	1.82-2.67		
**Severity of bodily pain**				
none	1.00		1.00	
mild	1.77	1.40-2.25	1.54	1.19-1.98
moderate	2.33	1.80-3.02	1.66	1.23-2.23
severe	3.78	2.85-5.02	2.35	1.68-3.28
**Consultation of a family doctor in the last 12 months**				
no	1.00		1.00	
yes	2.80	2.09-3.76	1.43	1.03-1.99
**Consultation of a specialist in the last 12 months**				
no	1.00		1.00	
yes	2.74	2.26-3.32	2.12	1.71-2.63
**Perceived social support (Oslo-3 Social Support Scale)**				
strong support	1.00		1.00	
intermediate support	1.44	1.18-1.74	1.39	1.04-1.84
poor support	1.62	1.24-2.12	1.23	1.01-1.51
				

*Abbreviations: OR – odds ratio, CI – confidence interval.

Age, marital status, region, education level, self-perceived general health, self-reported chronic diseases/conditions, bodily pain, consultation with a GP or a specialist in the last 12 months, and self-perceived social support were all significantly associated with unmet needs due to issues with transportation. After controlling for other factors in a multiple logistic regression model, the factors that remained significantly associated with unmet needs due to problems with transportation were region, education level, self-perceived general health, bodily pain, and self-perceived social support. Respondents living in the Adriatic region had higher adjusted odds ratio for unmet needs due to issues with transportation compared with those living in the Continental region. The same was true for those with only primary education compared with those with secondary education. Respondents with bad self-perceived general health, severe bodily pain, and poor self-perceived social support had a higher adjusted odds ratio for unmet needs due to problems with transportation (Table 5).

**Table 5:** Self-perceived unmet health care needs due to problems with transportation

	Self-perceived unmet health care needs due to problems with transportation			
Variable	OR unadjusted	95% CI	OR multivariable-adjusted**	95% CI
**Sex**				
male	1.00			
female	1.28	0.97-1.70		
**Age (years)**				
15-34	1.00			
35-44	1.54	0.78-3.03		
45-54	2.07	1.10-3.88		
55-64	1.47	0.82-2.64		
65-74	3.00	1.74-5.19		
75+	3.10	1.80-5.35		
**Marital status**				
never married and never in a registered partnership	1.00			
married or in a registered partnership	1.03	0.66-1.60		
widowed or in registered partnership that ended with death of partner	2.03	1.25-3.29		
divorced or in registered partnership that was legally dissolved	1.49	0.72-3.06		
**Region**				
Continental	1.00		1.00	
Adriatic	1.44	1.09-1.91	1.83	1.36-2.45
**Place of residence**				
urban	1.00			
rural	0.99	0.76-1.29		
**Education**				
primary or less	1.00		1.00	
secondary	0.50	0.38-0.68	0.67	0.49-0.91
higher than secondary	0.54	0.35-0.83	0.92	0.58-1.46
**Net monthly equalized income of the household (quintiles)**				
1st (poorest)	1.00			
2nd	1.04	0.66-1.65		
3rd	0.94	0.56-1.56		
4th	0.63	0.38-1.05		
5th (richest)	0.75	0.43-1.30		
**Self-perceived general health**				
very good and good	1.00		1.00	
fair	2.39	1.67-3.41	2.01	1.35-2.99
bad and very bad	5.42	3.73-7.86	3.58	2.28-5.61
**Self-reported chronic diseases/conditions**				
no	1.00			
yes	2.09	1.50-2.90		
**Severity of bodily pain**				
none	1.00		1.00	
mild	1.54	1.00-2.39	1.20	0.77-1.86
moderate	2.10	1.35-3.30	1.13	0.71-1.81
severe	5.25	3.33-8-25	2.39	1.43-3.99
**Consultation of a family doctor in the last 12 months**				
no	1.00			
yes	1.99	1.29-3.06		
**Consultation of a specialist in the last 12 months**				
no	1.00			
yes	1.67	1.24-2.24		
**Perceived social support (Oslo-3 Social Support Scale)**				
strong support	1.00		1.00	
intermediate support	1.39	1.00-1.92	2.03	1.36-3.05
poor support	2.42	1.65-3.56	1.32	0.95-1.83

*Abbreviations: OR – odds ratio, CI – confidence interval.

Age, urban/rural place of residence, self-perceived general health, self-reported chronic diseases/conditions, bodily pain, and self-perceived social support were significantly linked to unmet needs due to financial reasons. After controlling for other factors using a multiple logistic regression, the variables that remained significantly associated with unmet needs due to financial reasons included region, urban or rural residence, self-perceived general health, bodily pain, and self-perceived social support. Individuals living in the Adriatic region had a higher adjusted odds ratio for unmet needs due to financial reasons compared with those residing in the Continental region. Rural dwellers had a lower adjusted odds ratio for unmet needs compared with urban dwellers. Respondents with bad self-perceived health, severe bodily pain, and poor self-perceived social support had a higher adjusted odds ratio for unmet needs due to financial reasons (Table 6).

**Table 6:** Self-perceived unmet health care needs due to financial reasons

	Self-perceived unmet health care needs due to financial reasons			
Variable	OR unadjusted	95% CI	OR multivariable-adjusted*	95% CI
**Sex**				
male	1.00			
female	1.23	1.00-1.52		
**Age (years)**				
15-34	1.00			
35-44	1.48	0.97-2.25		
45-54	1.72	1.14-2.60		
55-64	1.46	1.03-2.90		
65-74	1.71	1.20-2.42		
75+	2.12	1.50-2.99		
**Marital status**				
never married and never in a registered partnership	1.00			
married or in a registered partnership	1.14	0.86-1.51		
widowed or in registered partnership that ended with death of partner	1.33	0.95-1.88		
divorced or in registered partnership that was legally dissolved	1.44	0.85-2.43		
**Region**				
Continental	1.00		1.00	
Adriatic	1.17	0.95-1.44	1.34	1.07-1.67
**Place of residence**				
urban	1.00		1.00	
rural	0.63	0.52-0.77	0.63	0.51-0.77
**Education**				
primary or less	1.00			
secondary	0.82	0.66-1.03		
higher than secondary	0.83	0.60-1.14		
**Net monthly equalized income of the household (quintiles)**				
1st (poorest)	1.00			
2nd	0.90	0.65-1.26		
3rd	0.95	0.67-1.34		
4th	0.98	0.69-1.41		
5th (richest)	0.88	0.60-1.32		
**Self-perceived general health**				
very good and good	1.00		1.00	
fair	1.49	1.17-1.89	1.26	0.96-1.65
bad and very bad	1.86	2.19-3.75	2.04	1.46-2.84
**Self-reported chronic diseases/conditions**				
no	1.00			
yes	1.56	1.26-1.93		
**Severity of bodily pain**				
none	1.00		1.00	
mild	1.83	1.39-2.41	1.67	1.24-2.24
moderate	2.18	1.63-2.92	1.62	1.16-2.27
severe	3.46	2.53-4.73	2.33	1.59-3.41
**Consultation of a family doctor in the last 12 months**				
no	1.00			
yes	1.21	0.90-1.64		
**Consultation of a specialist in the last 12 months**				
no	1.00			
yes	1.16	0.94-1.43		
**Perceived social support (Oslo-3 Social Support Scale)**				
strong support	1.00		1.00	
intermediate support	1.34	1.06-1.70	1.46	1.08-1.98
poor support	1.74	1.30-2.34	1.19	0.93-1.52

*Abbreviations: OR – odds ratio, CI – confidence interval.

## DISCUSSION

Several sociodemographic factors, such as the level of education, urban-rural residence, and living in the Adriatic Region were associated with a higher risk for UHN, but the association varied depending on the cause of UHN. A worse self-perceived health status, higher perceived levels of bodily pain, consultation with a physician in the last 12 months, and lower perceived levels of social support were connected to increased odds for unmet health needs.

The patterns of UHN observed in Croatia both align with and differ from findings in other European countries covered by EHIS. While rural residence is often linked with greater health care access barriers (11,12), in our study, rural residents had a lower odds of unmet needs due to financial and waiting-time-related reasons. One possible explanation is that rural residents may have stronger social support networks, which reduces their perceived unmet needs. Another explanation is that UHN are self-perceived, and rural residents may have lower health care expectations or higher satisfaction with the health care system (13). Additionally, these discrepancies might be affected by variations in health care system organization, geographic accessibility, and cultural expectations regarding health care use, which warrants further investigation.

In our study, sex was not significantly linked with unmet health care needs in either descriptive statistics or multivariate logistic regression models. This aligns with the findings from some studies (11,14-17). However, other researchers have reported sex-based disparities (12,18-20). A possible explanation is that women, particularly older women, have limited access to health care because they are less likely to have a driver’s license, especially in regions with inadequate public transportation. This factor should be explored in future studies on gender disparities in health care access.

Living in the Adriatic Region was linked to UHN due to transportation and financial difficulties, a finding emphasizing the need for targeted interventions to improve health care accessibility in this area. Given that 9.4% of the Adriatic region’s population lives on islands (21), geographic constraints likely contribute to these disparities. Prior studies have confirmed a strong link between transportation difficulties and UHN, even after adjustment for other factors (22).

Higher life satisfaction and community-based assistance could mitigate access barriers, providing informal solutions to health care challenges. This aligns with studies highlighting the effects of social cohesion in rural settings, which may compensate for systemic health care shortcomings (13). Lower levels of social support were strongly associated with all three aspects of UHN, which emphasizes the role of social networks in accessing health care. A lack of social support has been linked to adverse health outcomes and increased barriers to care (23), a finding highlighting the importance of strengthening social networks within communities.

Education was associated with UHN in a complex manner. Higher odds of UHN due to long waiting times were observed among respondents with higher education, while lower odds of unmet needs due to transportation issues were observed among respondents with secondary education than among those with primary education. Previous studies have reported mixed results regarding education and UHN. Some found no association (7,11,14) while others linked lower education levels with higher UHN (12,17,19). These inconsistencies could derive from differing expectations of health care services, with individuals with higher education levels being more aware of health care shortcomings and more likely to report unmet needs.

In our study, income was not significantly linked with UHN, which differs from findings in several previous studies (15,20,24-26). This discrepancy may be attributed to Croatia’s universal health care system, which reduces direct financial barriers to access. However, differences in previous occupations could still influence health care needs and access. Physically demanding jobs may lead to greater health care needs, while individuals from lower-income backgrounds might underreport unmet needs due to lower expectations of care. Future research should explore whether occupational histories shape health care-seeking behaviors in Croatia.

Health care use, represented by consultations with a family doctor or specialist in the last 12 months, was associated with an increased odds of reporting unmet needs due to long waiting times. However, no association was observed with unmet needs due to transportation or financial reasons. This suggests that frequent health care users may experience more frustration with system inefficiencies, rather than financial or geographic barriers. Similar findings have been reported in previous studies (14).

Self-reported chronic disease/condition was linked to all three aspects of unmet health care needs in univariate analysis but not in multivariate models. On the other hand, self-perceived general health and severity of bodily pain remained significant in the multivariate model, suggesting that self-perceived health status plays a more direct role in shaping health care access experiences. These findings are consistent with previous research (13,19).

A key limitation in survey-based studies is selection bias. Individuals with severe health conditions or mobility limitations may be underrepresented in surveys like EHIS, which leads to the underestimation of UHN. Additionally, these data sets do not capture respondents with extreme unmet needs that result in severe health deterioration or premature death. This could partly explain why some determinants, such as income and marital status, did not remain significant in the multivariate analysis, despite being linked to unmet needs in univariate analysis. Other studies have also reported that survey-based research tends to exclude individuals with the highest levels of health care needs (12,15,27). A more comprehensive assessment of unmet needs should consider complementary data sources such as hospital admission records and qualitative interviews with high-risk populations. This study’s reliance on self-reported data introduces subjectivity in assessing UHN. Differing perceptions of health needs and barriers across cultural and individual contexts may affect cross-country comparisons. Additionally, self-reported data on chronic conditions, bodily pain, and health care utilization may be influenced by recall bias, which should be considered when interpreting the findings.

Understanding the structural and personal determinants of UHN is crucial for shaping effective public policies. Our findings suggest that addressing health care accessibility in the Adriatic region, improving transportation infrastructure, and strengthening social support networks could mitigate unmet needs. Additionally, as private health care services expand in Croatia, future research should examine their impact on UHN and equity in health care access.
